# Antibodies Covalently Immobilized on Actin Filaments for Fast Myosin Driven Analyte Transport

**DOI:** 10.1371/journal.pone.0046298

**Published:** 2012-10-03

**Authors:** Saroj Kumar, Lasse ten Siethoff, Malin Persson, Mercy Lard, Geertruy te Kronnie, Heiner Linke, Alf Månsson

**Affiliations:** 1 School of Natural Sciences, Linnaeus University, Kalmar, Sweden; 2 The Nanometer Structure Consortium and Division of Solid State Physics, Lund University, Lund, Sweden; 3 Department of Women’s and Children’s Health, University of Padua, Padova, Italy; University of California, Merced, United States of America

## Abstract

Biosensors would benefit from further miniaturization, increased detection rate and independence from external pumps and other bulky equipment. Whereas transportation systems built around molecular motors and cytoskeletal filaments hold significant promise in the latter regard, recent proof-of-principle devices based on the microtubule-kinesin motor system have not matched the speed of existing methods. An attractive solution to overcome this limitation would be the use of myosin driven propulsion of actin filaments which offers motility one order of magnitude faster than the kinesin-microtubule system. Here, we realized a necessary requirement for the use of the actomyosin system in biosensing devices, namely covalent attachment of antibodies to actin filaments using heterobifunctional cross-linkers. We also demonstrated consistent and rapid myosin II driven transport where velocity and the fraction of motile actin filaments was negligibly affected by the presence of antibody-antigen complexes at rather high density (>20 µm^−1^). The results, however, also demonstrated that it was challenging to consistently achieve high density of functional antibodies along the actin filament, and optimization of the covalent coupling procedure to increase labeling density should be a major focus for future work. Despite the remaining challenges, the reported advances are important steps towards considerably faster nanoseparation than shown for previous molecular motor based devices, and enhanced miniaturization because of high bending flexibility of actin filaments.

## Introduction

The imperative to continue ensuring human health and welfare, as well as animal health, demands improvement of bio-analytical methods, such as the introduction of miniaturized biosensors with increased sensitivity, rate of detection and capacity for multiplexing [1], [2], [3]. To that end, nanostructure-based devices [1], [4], [5], [6], [7] have been developed and are often combined with microfluidics based separation and concentration schemes [2], [3], [8]. However, together with accessory infrastructure, microfluidic devices exhibit appreciable complexity and bulkiness [2], [8] or require strong driving forces for liquid transport in nanofluidics [9]. These critical issues might be circumvented by the use of ATP-driven biological molecular motors for separation and concentration of analytes (e.g a protein or oligonucleotide biomarker) [10]. In such an approach, microfluidic pumps and other artificial components (e.g. nanoparticles) are substituted by highly miniaturized and energy efficient molecular motor driven propulsion of cytoskeletal filament shuttles [11]. One may envisage that the filaments, with bound recognition molecules (antibodies, oligonucleotides), capture analytes from solution, [12], [13], [14], [15], [16], [17], [18], [19] thus replacing inorganic particles often used for this purpose [1]. Next, the filaments bind specifically to predetermined surface areas where active molecular motors are adsorbed. Upon addition of ATP, the filaments are then propelled along nanostructured channels/tracks [9], [10], [11], [14], [20], [21], [22], [23], [24], [25] for filament and analyte concentration to a detector site. The feasibility of this approach has recently been demonstrated in proof-of-principle devices [26], [27] using the kinesin-microtubule motor system and micropatterned surfaces. However, due to the inherently low speed of the kinesin-microtubule system, the devices exhibited rather slow separation and detection compared to existing high-sensitivity methods [5], [28], [29], [30]. In addition, the antibody-conjugation of the microtubules using biotin-streptavidin links have critical limitations compared to covalent immobilization of antibodies to filaments [15], [31].

An attractive solution to overcome slow separation is the use of myosin driven propulsion of actin filaments. This offers motility one order of magnitude faster than the kinesin-microtubule system when fast myosin II from skeletal muscle is used. The actin filaments also have lower flexural rigidity than the microtubules facilitating further miniaturization. However, covalent conjugation of actin filaments with antibodies for antigen capture and transportation has not yet been reported. This may be attributed to some challenging properties of actomyosin that are precisely the properties underlying the mentioned benefits of this motor system. Thus, due to the comparatively low actin affinity of myosin II motors (pre-requisite for high speed), and the low number of myosin binding sites per µm of the actin filament length (related to the high bending flexibility), one can foresee increased likelihood that antibody conjugated actin filaments detach from the surface (see [10], [21], [32]). Additionally, since actin filaments have been suggested to rotate around their long axis during motility [33] this may result in collisions between antibody-antigen cargo and the dense motor layer [10]. A final concern in the use of covalently antibody conjugated actin filaments is raised by observations [34], [35], [36], [37], [38] where, for instance, binding of certain proteins to actin filaments alter filament structure and where covalent modifications of actin monomers interfere with motility [39], [40]. Microtubules seem to be more robust in this regard [41].

We here report results showing that the above expressed concerns on use of the actomyosin system for miniaturized biosensors have been largely unfounded. A key result is the realization of a novel approach for covalent antibody-attachment to cytoskeletal filaments using heterobifunctional cross-linkers [42], [43], [44]. Unlike the cross-linkers used previously for attachment of antibodies to microtubules [15], the heterobifunctional linkers allow attachment of both poly and monoclonal antibodies to the filaments. This versatility is essential to any state-of-the-art biosensing system aiming at maximal specificity and sensitivity in analyte detection. Moreover, a key advancement of our results is the first achievement of covalent attachment of antibodies to actin filaments without interfering appreciably with actomyosin motility. These results open the door to exploiting the advantages of the actomyosin system in biosensor development, in particular high speed and great potential for miniaturization. In addition, we discuss the need of achieving consistently high conjugation-ratio (antibody/actin monomer) as an important challenge for further development towards biosensor applications. In our concluding discussion, we consider pros and cons of actin filaments, actin filament bundles [21], [32] and kinesin driven microtubules as shuttles for transportation of antibody-antigen complexes in biosensing nanodevices.

## Materials and Methods

### Materials

C6-succinimidyl 6-hydrazinonicotinate acetone hydrazone (C6-SANH), C6-succinimidyl 4-formylbenzoate (C6-SFB; see further list of abbreviations in Abbreviations S1), 2-sulfobenzaldehyde (2-SBA) and 2-hydrazinopyridine.dihydrochloride (2-HP) were purchased from Solulink, San Diego, CA, USA. Anti-rabbit IgG (H&L, Goat, a-rIgG) was purchased from Rockland Immunochemicals, Gilbertsville, USA. Anti-human CD45 monoclonal antibody (Clone: T29/33) was purchased from Universal Biologicals LTD, Cambridge, UK. Tetramethylrhodamine-isothiocyanate (TRITC) labeled goat anti-mouse IgG (2–8 fluorophores/IgG), Novex Tris-glycine pre cast gels (10%) and molecular marker (SeeBlue® Plus2) were purchased from Invitrogen, Stockholm, Sweden. Streptavidin labeled with TRITC, zeba desalt spin columns, bicinchoninic acid (BCA) protein assay kits, EZ-Link ^TM^Sulfo-NHS-LC-biotinylation kits and *N*-Hydroxysuccinimide-Rhodamine (NHS-Rh) were purchased from Pierce Rockford, IL, USA. Rabbit IgG (rIgG) and all other chemicals were of analytical grade and, unless otherwise stated, purchased from Sigma-Aldrich Sweden AB, Stockholm, Sweden.

### Biotinylation and Fluorescence Labeling of Rabbit IgG

The rIgG (2 mg mL^−1^) was incubated with EZ-Link Sulfo-NHS-LC-biotin for 2 h at room temperature (RT) followed by purification with zeba desalt spin column. The extent of biotinylation was determined by 4′-hydroxyazobenzene-2-carboxylic acid (HABA) assay to 4.5 biotins per IgG. For labeling of rIgG with rhodamine, 10 fold molar excess of NHS-Rh (dissolved in dimethylsulfoxide, DMSO) was incubated with 2 mg mL^−1^ of rIgG in 0.1 M phosphate buffered saline (PBS; 0.1 M sodium phosphate, 0.15 M NaCl, pH 7.4) for 2 h at RT in the dark and then purified by zeba desalt spin column. The average extent of labeling was spectrophotometrically determined as ∼1.9 rhodamines per rIgG, by measuring absorbance at 280 nm and 555 nm using an UV-vis spectrophotometer (Shimadzu UV-1800; Kyoto, Japan). The molar extinction coefficient of rhodamine and rabbit IgG was taken as 80,000 M^−1^ cm^−1^ (555 nm) and 210,000 M^−1^ cm^−1^ (280 nm), respectively.

### Modification of F-actin with C6-SANH

Free amine containing contaminants from F-actin solutions were removed using ultracentrifugation (Beckman-Coulter airfuge, 100,000 g, 45 min) through a 10% glycerol cushion. Pellets were resuspended in modification buffer 1∶100 mM ((4-(2-hydroxyethyl)-1-piperazineethanesulfonic acid) (HEPES), 150 mM KCl, 5 mM MgCl_2_, 1 mM Na_2_ATP, pH 8.0) with 5% glycerol. After storage on ice (30 min) a second ultracentrifugation step was applied with subsequent resuspension in modification buffer. After storage on ice for 20 min the actin solutions were sonicated gently on ice to disperse the filaments. F-actin (3.5 mg mL^−1^) was incubated separately with C6-SANH linker (dissolved in DMSO) at RT for 2 h with molar ratios 1∶1, 1∶2 or 1∶3 (actin:linker). Excess linker was removed by two air centrifugation washes (see above) followed by resuspension in conjugation buffer: 100 mM (2-(*N*-morpholino)ethanesulfonic acid (MES), 150 mM KCl, 5 mM MgCl_2_, 1 mM Na_2_ATP, pH 6.0) and subsequent storage on ice.

### Quantification of C6-SANH Incorporation into F-actin

Modified F-actin samples (10 µL of 80 µM) were mixed into 490 µL of 2-SBA (dissolved in DMSO and diluted to 0.5 mM in MES buffer (100 mM MES, pH 5.0)) solution and incubated for 1 h at 37°C. A blank sample was prepared as above but with 10 µL of conjugation buffer instead of modified actin sample. After incubation, 10 µL of 10 M NaOH was added to each reaction sample. The absorbance attributed to the bis-aryl hydrazone bond that formed between 2-SBA and C6-SANH modified actin, was then measured at 350 nm. The molar substitution ratio of each reaction was calculated using the Solulink online protein modification calculator (http://www.solulink.com/support/protocols).

### Modification of Antibodies with C6-SFB

The a-rIgG (polyclonal antibody) solution was exchanged with modification buffer 2 (0.1 M sodium phosphate, 0.15 M NaCl, pH 7.4) by zeba desalt spin column to remove free amine base contaminants. The a-rIgG solution (2 mg mL^−1^) was then incubated separately with 5 or 10-fold molar excess of C6-SFB linker (dissolved in DMSO) at RT for 2 h. Modified a-rIgG was desalted against conjugation buffer (see above) to remove excess linker and to exchange buffer. The modified antibodies were kept on ice until the conjugation reaction with modified actin. Anti-human CD45 monoclonal antibody (3.4 mg mL^−1^) was modified as above using 10-fold molar excess of C6-SFB linker.

### Quantification of C6-SFB Incorporation into Antibodies

A solution of 2-HP was diluted in MES buffer to a final concentration of 0.5 mM. The formation of the bis-aryl hydrazone bond between 2-HP and C6-SFB modified antibodies was then verified as follows. First, 10 µL of 13 µM C6-SFB modified antibody samples were mixed with 490 µL of the diluted 2-HP solution and incubated for 1 h at 37°C. After incubation, 10 µL of 10 M NaOH was added to each reaction sample. Absorbance was measured at 350 nm and the molar substitution ratio was calculated using the Solulink online protein modification calculator. A blank sample was incubated as above but with conjugation buffer instead of modified antibody.

### Conjugation of F-actin to Anti Rabbit IgG and Anti-human CD45 Antibody

Three different C6-SANH modified F-actin samples (1, 2 and 3 molar excess of C6-SANH linker) were incubated with the two different C6-SFB modified a-rIgG solutions (5 and 10-fold molar excess of C6-SFB linker). Addition of 10 mM catalyst buffer from stock solution (100 mM aniline in conjugation buffer) started the reaction that was allowed to proceed for 6 h at RT. In each conjugation reaction the molar ratio of modified F-actin and modified a-rIgG was 2∶1 respectively. Thus, according to the different modification ratios, five different conjugation samples (Ac1-Ac5) were obtained as follows: Ac1) F-actin modification 1∶1 and a-rIgG modification 1∶10, Ac2) F-actin modification 1∶2 and a-rIgG modification 1∶10, Ac3) F-actin modification 1∶1 and a-rIgG modification 1∶5, Ac4) F-actin modification 1∶2 and a-rIgG modification 1∶5, and Ac5) F-actin modification 1∶3 and a-rIgG modification 1∶5. Sample names and modification ratios are summarized in Tables S1–S2. For conjugation of F-actin to anti-human CD45, monoclonal antibody (MAb; antibody from mouse), F-actin (actin:linker ratio, 1∶2) and anti-human CD45 antibody (antibody:linker ratio, 1∶10) was incubated as above (AcM; Table S2).

In order to remove catalyst and change the buffer, samples were dialyzed for 38 h at 4°C against G-actin buffer (2 mM Tris base, pH 8.5, 0.2 mM Na_2_ATP, 0.5 mM dithiothreitol (DTT), 0.2 mM CaCl_2_, 3 mM NaN_3_) or centrifuged (Beckman-Coulter airfuge, 100,000 g, RT, 60 min or Optimamax-XP ultracentrifuge, 100,000 g, 4°C, 30 min). After centrifugation, pellets were re-suspended either in G-actin buffer (“Filament Formation Method 1”) or F-actin buffer (G-actin buffer with 60 mM KCl, 2 mM MgCl_2_, 3.3 mM ATP; “Filament Formation Method 2”). In the latter method, pellets were homogenized in F-actin buffer and gently sonicated on ice to disperse the filaments. Actin filaments were then labeled with Alexa-488 phalloidin (APh) (molar ratio: 1∶1.5; actin:APh) at 4°C over night. For long-term storage, samples were flash frozen in liquid nitrogen and stored at −80°C.

In Filament Formation Method 1, the conjugated G-actin monomers (actin-a-rIgG) were co-polymerized with non-conjugated monomers to form filaments. Poor polymerization was observed for high ratios (conjugated:non-conjugated) whereas too low ratios made it difficult to detect the conjugates by fluorescent r-IgG. As a suitable compromise we used a 1∶3 (conjugated:non-conjugated) ratio for in vitro motility assay (IVMA) experiments. The co-polymerization reaction was performed at 4°C (4 h) by addition of KCl, MgCl_2_ and ATP to final concentrations of 100 mM, 2 mM and 3.3 mM, respectively. The co-polymer was fluorescence-labeled with APh (molar ratio: 1∶1.5; actin:APh) at 4°C over night.

### Antigen Binding to Antibody Conjugated Actin Filaments

After 5 h of APh labeling in solution, biotinylated rIgG (BT-rIgG) (or rhodamine labeled rIgG (Rh-rIgG)) was added to final concentrations of 10–500 nM and incubated for 8 h. For the biotin-streptavidin system, 1 to 50 nM of TRITC labeled streptavidin was added approximately one hour prior to motility experiments. The approach was similar for the system with monoclonal antibodies but in this case anti-mouse-IgG, rather than antigen was used for detection of antibody labeling. Thus, after 5 h of APh labeling in solution, TRITC-anti-mouse IgG was added to final concentration of 100–250 nM and incubated for 8 h. In one set of control experiments a-rIgG conjugated actin filaments were instead first immobilized to HMM on an in vitro motility assay surface before incubation with 10 nM rabbit IgG for 60 min followed by addition of assay buffer for observation of motility.

### Analysis of Actin-antibody Conjugation using SDS-PAGE

Conjugation reaction samples were run on a Novex® 10% Tris-Glycine gel with Novex® Tris-Glycine SDS running buffer (Invitrogen) based on the Laemmli method under reducing conditions. Image J (Rasband, W.S ImageJ, U.S. National Institutes of Health, Bethesda, Maryland, USA, http://imagej.nih.gov/ij/, 1997–2012) Gel Analysis tool was used to analyze the SDS-PAGE gels in order to calculate an approximate conjugation yield. All bands on the gel showing higher molecular weight than the antibody heavy chain were considered to represent conjugation products. The area of the intensity peak for each individual band was measured as percent of the total intensity peak area on the gel. The amount of protein in µg was then calculated from a standard with known amount of actin. Also, the apparent molecular weight for each band was calculated using a protein standard (22–250 kDa) where the retardation and the logarithm of the molecular weight were fit to a second-order polynomial function. The molecular weights were then used to obtain the relative molar concentrations of conjugation products.

Because bands for the conjugated samples had rather low intensity and were somewhat overlapping (for higher order conjugates), they were lumped together as one band in the analysis (“conjugated actin”). The molecular weight for this band was calculated as the mean of the measured molecular weights giving average values of 110 kDa for Ac1-Ac3 and 125 kDa for Ac4 and Ac5 conjugates. The conjugation yield of the reaction was calculated by dividing the total concentration of the conjugation products with the total concentration of actin in the sample (assuming 1 actin monomer per conjugation product).

### Confirmation of Actin-antibody Conjugation Using Absorbance Spectroscopy

The formation of the bis-aryl hydrazone bond between C6-SANH modified actin and C6-SFB modified antibodies was verified by measuring absorbance using a UV-VIS Nano Drop Spectrophotometer (ND-100) at 350 nm (optical path length, 1 mm). In order to make absorbance data comparable to data for a conventional absorbance spectrophotometer (1 cm path length), absorbance values were multiplied by 10 before plotting. The blank sample contained conjugation buffer with catalyst. A mixture of actin and antibody at similar concentrations as in the conjugation reaction was used as a control.

### Fluorescence Microscopy Based Estimation of Rh-rIgG Binding to Actin Filaments

**Figure 1:**
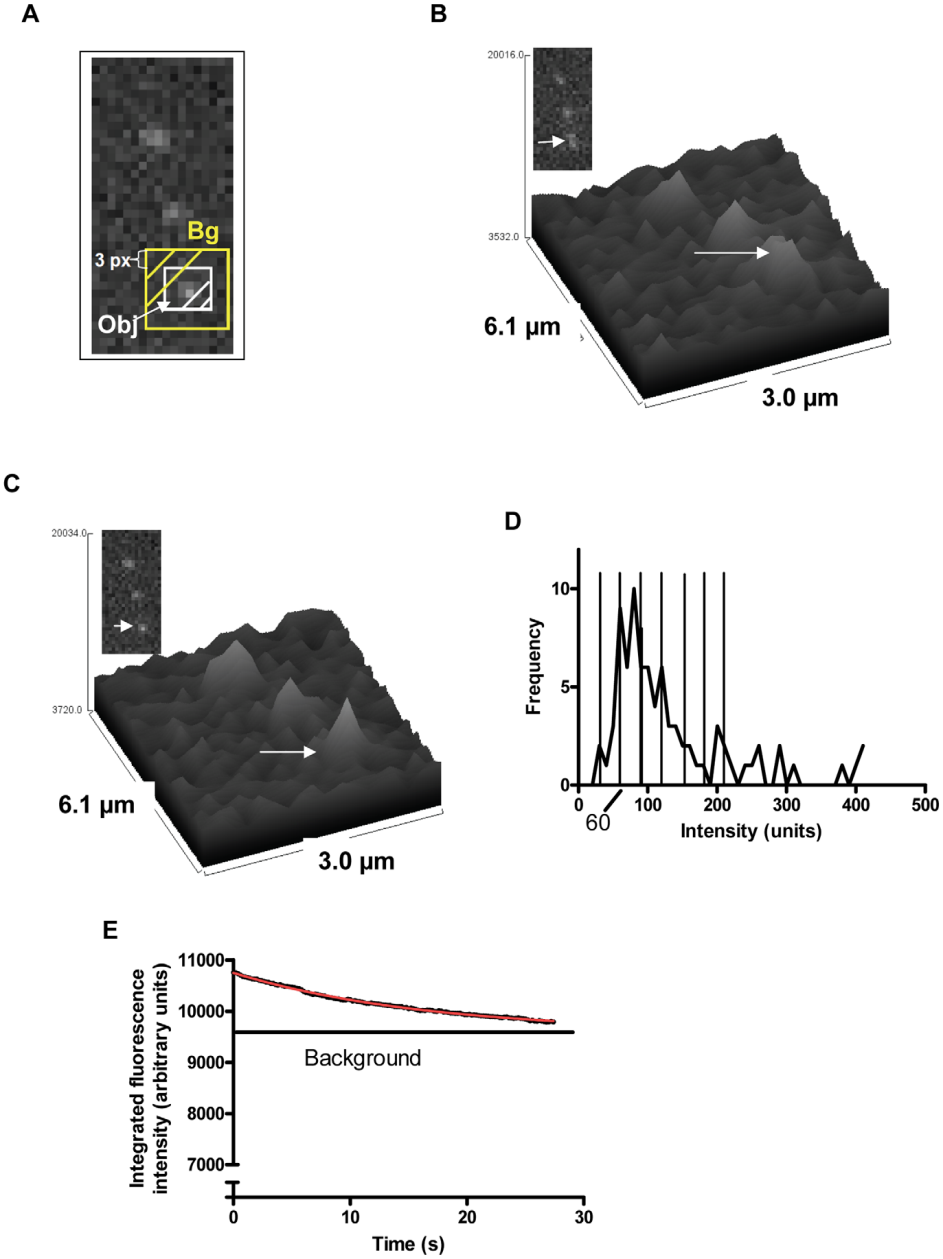
Fluorescence intensities attributed to Rh-rIgG after complex-formation with (a-rIgG) conjugated actin filaments. Observation using epi-fluorescence microscopy (TRITC filter set) and an EM-CCD camera with high gain after binding of the filaments (in the absence of ATP) to HMM on a surface. (**A**) Regions of interest “Bg” (yellow rectangle) and Obj (white rectangle) used to estimate background-subtracted intensity of diffraction limited fluorescent spot as described in the text. The Bg-rectangle is 3 pixels (0.165 µm/pixel) wider than Obj-rectangle. Same data as inset of Fig. 1C. (**B**) Surface plot illustrating spatial intensity distribution attributed to four fluorescent spots (inset) along one actin filament. The spot indicated by the arrow, exhibited photo-blinking, and is “on” in this image frame. (**C**) Same filament as in (A) observed 0.6 s later with disappearance of fluorescence spot at arrow due to blinking. Total observed drop in intensity: 70 intensity units (IntU). (**D**) Frequency polygon (bin width: 10 IntU) for fluorescence intensities of apparently isolated fluorescent spots (in total 87, observed in the given experiment). Vertical straight lines separate 30 IntU suggested to represent a single fluorophore as argued in the text. (**E**) Photobleaching of Rh-rIgG. The decay in fluorescence intensity during an observation period integrated over the entire image with Rh-rIgG bound to a-rIgG on actin filaments and non-specifically on the surface. The background level was obtained from fit (red line) of single exponential decay function (decaying to background level >0) to experimental data (black curved line).

The number of rhodamine or TRITC molecules in a region of an image (8-bit) was estimated from the background-subtracted fluorescence intensity using a Matlab program (Fig. 1A). The integrated intensity of a rectangular region of interest (ROI) fully enclosing the diffraction limited image of the fluorescent object (Obj, white; Fig. 1A) was first measured. This was followed by background subtraction based on pixel values in a surrounding ROI made up of the area between the large (“Bg”, yellow) and small (“Obj”, white) rectangle in Fig. 1A. The background-subtracted intensity, I, was then calculated using Equation 1.
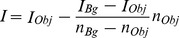
(1)


Here, the quantities I_Obj_ and I_Bg_ are the integrated intensity over the “Obj” and “Bg” ROIs, respectively (Fig. 1A) whereas nPx_Obj_ and nPx_Bg_ is the number of pixels in the “Obj” and “Bg” ROIs, respectively. A similar approach was used to obtain intensity values both for single fluorescent spots and for more extended objects, e.g. with several Rh-rIgGs along a filament. The experimental uncertainty (standard deviations) in the intensity values between measurements varied with the number of fluorophores as described in Table S3 and Fig. S1.

In order to obtain the intensity per Rh-rIgG, we first determined the fluorescence intensity attributed to apparent single rhodamine fluorophores. Particularly, we focused on those fluorophores that were attached to actin filaments (see Fig. 1A–C), as verified by HMM induced motility or co-localization with actin filaments co-stained with APh. In Fig. 1B–C it can be seen how one observed rhodamine spot photo-blinked (i.e. switched off temporarily) suggesting that it either represented a single fluorophore or that its fluorescence intensity was temporarily hidden in the background noise by the blinking of one of two fluorophores. By observing 87 fluorescent spots, in one experiment, we obtained the intensity distribution, (Fig. 1D) with a main broad peak centered between 60 and 100 intensity units (IntU). One interpretation of the intensity distribution in Fig. 1D, would be that the intensity (60–70 IntU) represented by the center of the broad peak corresponds to one fluorophore. However, an alternative approach gave results in conflict with this view. In this alternative approach we divided the fluorescence intensity per unit length of non-motile actin filaments saturated with TRITC-phalloidin (TRITC-Ph) with the number of phalloidin binding sites (∼360 per µm) [38], [45]. The exposure time was reduced to 0.05s, ¼ of that (0.2 s) used to observe Rh-rIgG labeled actin filaments, in order to avoid saturation of the CCD and with a simple conversion to the exposure time of 0.2 s by multiplication with 4. The rationale for this approach is the strong binding of TRITC-Ph to actin filaments, the lack of self-quenching between neighboring TRITC-phalloidin molecules on the actin filament [46], [47] and the similarity in quantum yield and extinction coefficient of TRITC-Ph and the NHS-rhodamine (cf. Invitrogen/Molecular Probes web-site; http://www.invitrogen.com/site/us/en/home/brands/Molecular-Probes.html). This method suggested that the intensity per rhodamine molecule was 28.9±3.6 IntU; (mean ± SEM, n = 13 filaments), i.e. approximately half the value indicated by the location of the broad peak of the intensity distribution in Fig. 1D. It therefore seems reasonable to believe that this peak is actually attributed to the overlap of sub-peaks at mainly ∼60, ∼90 and ∼120 IntU, corresponding to 2, 3 and 4 fluorophores respectively (see Discussion S1). On basis of these findings, we are confident to state that 28.9±3.6 IntU corresponds to a single rhodamine molecule. This was similar to the pixel to pixel variability in background intensity (standard deviation, 30–40 IntU) consistent with a detection limit of two rhodamine molecules (∼60 IntU). It is important to emphasize that the value of 29 IntU per rhodamine is not affected by photobleaching, because this process reduces the number of fluorescent molecules but not their intensity (provided that the physicochemical environment, e.g. temperature, pH etc is constant). However, in contrast, the number of fluorophores in any sample decreases with time after onset of illumination. We therefore measured fluorescence intensities of Rh-rIgG-(a-rIgG) labeled filaments within 10 s after switching on illumination, This would limit underestimation of the true number of fluorophores to ∼50% as suggested by the half-life of the photobleaching induced decay of Rh fluorescence intensity (Fig. 1E).

The number of Rh-rIgG molecules was estimated by dividing the total fluorescence intensity (I, obtained as described above for single fluorescent spots; Eq.1, Fig. 1) by 1.9×29 IntU, using the average number (1.9) fluorophores per Rh-rIgG obtained in spectrophotometric analysis and the intensity per single fluorophore estimated as described above. The uncertainty in the average number of fluorophores varied with the number of Rh-rIgGs (n_Rh_) given by 1.9/√n_Rh_ on the assumption of a Poisson distribution. Now, considering a full error propagation analysis (Table S3), the uncertainty in the number of Rh-rIgG molecules estimated from the ratio I/(1.9×29) would vary from 1–3 for a low number of Rh-rIgG (<5) to about 6 for ∼20 Rh-rIgG. As a necessary consequence of these uncertainties one would not expect to obtain integers for the estimated number of fluorophores. Appreciable systematic errors introduced by the assumption of similar fluorescence intensity per rhodamine on IgG as for TRITC-Ph on actin are unlikely as considered further in the Discussion S1.

### Muscle Protein Preparations for in vitro Motility Assays

Myosin II was purified from rabbit fast skeletal muscle and heavy meromyosin (HMM) was prepared by digestion of myosin with α-chymotrypsin treated with the trypsin inhibitor N-α-tosyl-L-lysyl chloromethyl ketone (TLCK). Actin was isolated from rabbit skeletal muscle [48] and actin filaments were fluorescently labeled with APh or TRITC-Ph (Molecular Probes Invitrogen, Eugene, OR) [49], [50], [51]. All experiments using animal material were performed in accordance with national and EU-legislation and were approved by the Ethical Committee for Animal experiments (reference # 96–11 at Linköping, Sweden).

### Surface Preparation for in vitro Motility Assays

Trimethylchlorosilane (TMCS) derivatized substrates were prepared as described previously [52]. In brief, microscope cover slips (No. 0, 24×24 mm, Menzel-Glaser, Braunschweig, Germany) were first cleaned by immersion in piranha solution (70% H_2_SO_4_ and 30% H_2_O_2_) at 80°C for 5 min. *(Caution! Piranha solution is a highly corrosive acidic solution that can react violently with organic materials. Do not store in a closed container, and take appropriate safety precautions)*. Subsequent immersion steps were as follows (2 min each): i. deionized water (three times), ii. methanol, iii. dry acetone, and iv. dry chloroform. The slide was then dried (in N_2_ gas stream) and dipped in a freshly prepared TMCS solution (5% TMCS in chloroform). *Caution: TMCS is highly flammable and reacts violently with water!* Finally, the surfaces were dipped in dry chloroform twice, dried (N_2_) and stored under ambient conditions. Surfaces were rinsed and kept in deionized water for more than 30 min before they were dried and assembled into flow cells [52]. The properties of TMCS derivatized substrates were verified by measuring advancing and receding contact angles for water droplets as described previously [53].

### In vitro Motility Assays

Flow cells were constructed from two cover-slips with double-sided sticky tape as spacers [22], [50], [52]. The motility supporting surface (bottom of flow cell) was a TMCS-derivatized cover slip described above. All solutions that were added to the flow cell were based on buffer A (1 mM MgCl_2_, 10 mM 3-morpholinopropane-1-sulfonic acid (MOPS), 0.1 mM K_2_-ethylene glycol tetraacetic acid (EGTA), pH 7.4) and all proteins were diluted in buffer B (buffer A with 1 mM DTT and 50 mM KCl). The flow cell was pre-incubated according to standard procedures [22], [49], [50], [52]: (i). HMM (120 µg mL^−1^) for 2 min, (ii). 1 mg mL^−1^, bovine serum albumin (BSA) for 30 s, (iii). blocking actin (1 µM unlabeled sheared actin filaments with 1 mM MgAdenosine-5'-triphosphate (MgATP)) for 1 min (this step omitted in a majority of the experiments; see further under Results and Discussion). The mentioned pre-incubation steps were followed by (iv) wash with a50 assay solution (buffer A with 10 mM DTT, 1 mM MgATP, 35 mM KCl, ionic strength 50 mM) and (v) addition of antibody-conjugated or non-conjugated actin filaments at 10 nM–100 nM in buffer B. After an incubation period of 3 min, flow cells were washed with buffer B, and (vi) incubated with assay solution a60 or aMC130, having similar compositions as the a50 solution but with different KCl concentrations to obtain different ionic strengths of 60 and 130, respectively. At a high ionic strength, 0.64% methylcellulose was included (aMC130). Moreover, an ATP regenerating system (2.5 mM creatine phosphate and 3.5 U mL^−1^ creatine phosphokinase) was added, as well as an anti-bleach system containing final activity concentrations of 3 mg mL^−1^ glucose, 20 U mL^−1^ glucose oxidase and 870 U mL^−1^ catalase.

A Nikon Eclipse TE300 inverted fluorescence microscope (Nikon Corp., Tokyo, Japan) was used for the observation of actin filaments. The microscope was equipped with a temperature-regulated Nikon (100×1.4 NA) oil immersion objective, TRITC (Ex. 540/25, DM 565, and BA 605/25) and FITC (Ex. 465–495, DM 505, and BA 515–555) filter sets. Image sequences were recorded using a cooled Hammamtsu EMCCD camera as described previously [51].

Filament lengths, intensities and velocities were obtained using a Matlab based manual tracking program [54], [55], [56]. If not otherwise stated, sliding velocities were obtained during the 10-frame period with smoothest sliding (lowest coefficient of variation [CV] of the frame-to-frame velocity) during randomly selected 15–40 frame observation periods (frame rate: 5 s^−1^) and paths with CV >0.5 were excluded from analysis. Underestimation of velocities due to path truncation is negligible, ensured by the design of the analysis algorithm and manual exclusion of highly curved paths. The short but repeated observation periods as a basis for the velocity measurements ensured sampling of a large number of different filaments (cf. [57]). Considerably longer sequences of several hundred frames were also captured on each experimental occasion for prolonged observation of individual filaments.

The filament tracking program was supplied with a routine for measurement of filament length. Here, the length was obtained by application of the Pythagorean theorem, using coordinates selected to divide the filament into straight segments.

### Statistical Analysis

Statistical analyses including regression analyses were implemented in GraphPad Prism (v. 5.0; GraphPad Software, San Diego, CA). Unless otherwise stated, data are given as mean ± standard error of the mean (SEM) and statistical hypothesis testing was performed using two-tailed Student´s t-test (paired when appropriate).

## Results and Discussion

### Covalent Antibody-immobilization to Actin Filaments

**Figure 2:**
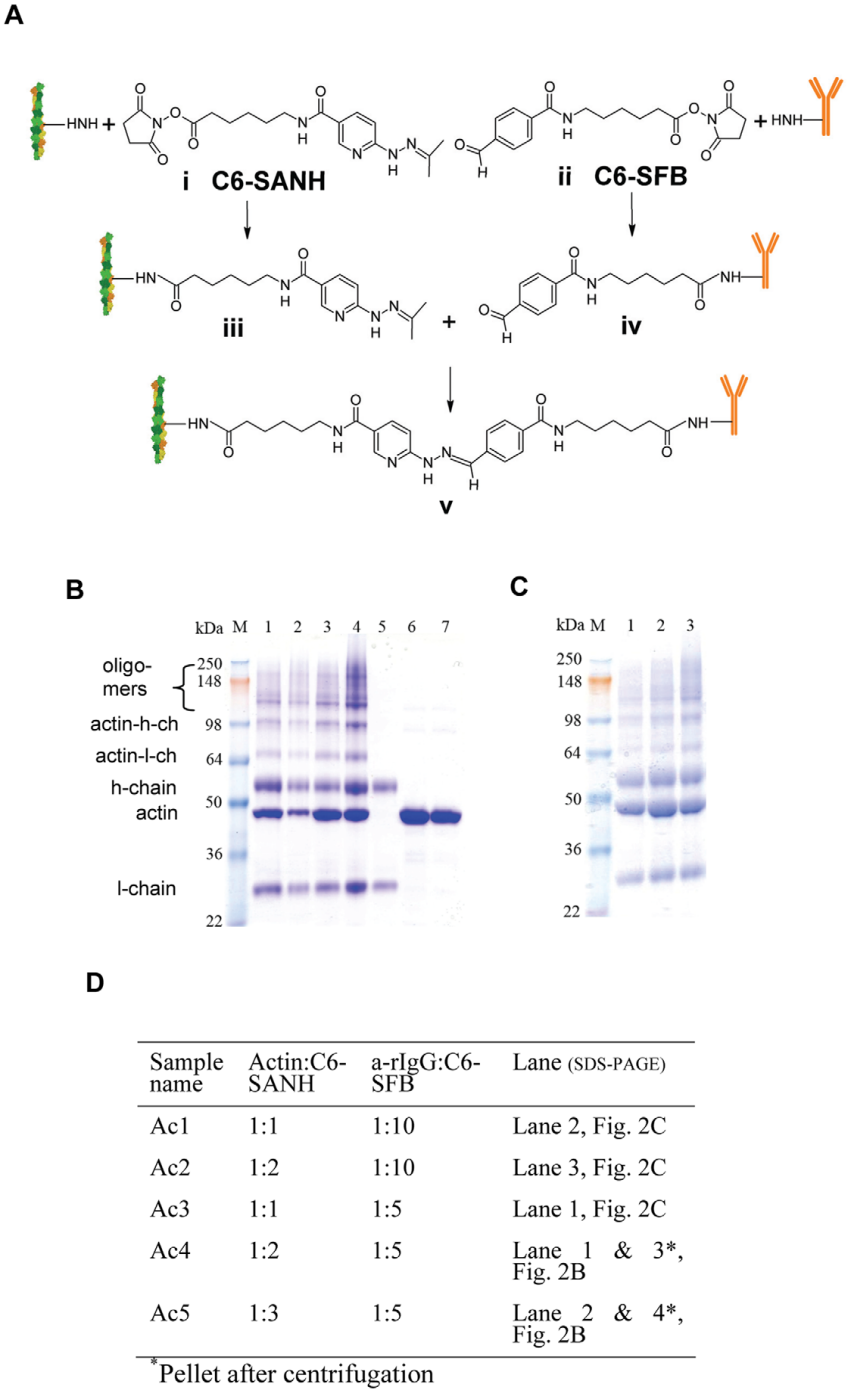
Covalent attachment of a-rIgG to actin monomer. (**A**) Principle. C6-SANH (i, iii) aromatic hydrazine protected as acetone hydrazone and C6-SFB (ii, iv) reacted with primary amines of lysines on actin filament and a-rIgG, respectively. Next (v), aromatic hydrazone modified actin filament reacted with aromatic aldehyde modified a-rIgG forming stable bis-aryl hydrazone bond. (**B**) Gel under reducing conditions (SDS-PAGE) of actin-(a-rIgG) conjugates. Lanes 1–4: Conjugates Ac4 and Ac5 (2 and 3 molar excess of C6-SANH over actin, respectively, and 5 molar excess of C6-SFB over a-rIgG). Lanes 1–2 and 3–4 represent conjugates prior to ultracentrifugation and pellet after ultra-centrifugation (containing conjugated actin in filament form; F-actin), respectively. Lane 5. a-rIgG modified with 10 fold molar excess of C6-SFB. Lanes 6–7: non-conjugated actin reacted with 2 and 3 molar excess C6-SANH, respectively. The first lane denoted “M”: molecular marker (SeeBlue Plus2®). Approximately 3 µg of proteins in each well. (**C**) Lanes 1–3: conjugated samples Ac3, Ac1 and Ac2. Actin reacted with 1 (Ac3 and Ac1) and 2 (Ac2) molar excess of C6-SANH respectively. Labeling of different bands to the left: l-chain (l-ch) and h-chain (h-ch) denote antibody light chain and heavy chain, respectively. (**D**) Overview of samples shown in different lanes of SDS-PAGES in B and C.

Our approach to achieve covalent antibody conjugation to actin filaments by reaction between the hetero-bifunctional cross-linkers (Fig. 2A) C6-SANH on actin and C6-SFB [42], [43], [44] on a-rIgG circumvented the formation of extensive actin-actin or antibody-antibody cross-linking. The latter problems are seen when homobifunctional linkers such as glutaraldehyde [13] are used. Moreover, the linkers do not cross-react with functional groups present in amino acids [42], [43], [58]. Attaching antibodies to cytoskeletal filaments via biotin-streptavidin linkages [12], [14], [26], [27], [59] (the most frequently used method) has been shown to be associated with extensive inter-filament cross-linking [14], [31] that requires [14] immobilization of the filaments on motor coated biosensor-surfaces before attachment of the recognition elements [26], [27]. This approach results in limited versatility e.g. difficulties to routinely analyze the degree of antibody conjugation without extracting the actin filaments and thereby destroying the device.

The C6-SANH linker on actin is likely to attach to one of the most accessible lysines (in order from most to least accessible: 1. Lys-113, 2. Lys 215 and 3. Lys-50, Lys-191, Lys-284, Lys-291, Lys-315, Lys-326 and Lys-359; analysis using Swiss-PdbViewer 4.0.4 (http://www.exapzy.org/spdbv/); Protein data bank entry 1M8Q). Importantly, covalent biotinylation of up to 5 of the lysines, using the same conjugation chemistry as for C6-SANH, was possible without noticeable effect on actomyosin motility, suggesting minimal changes in filament structure and function due to covalent modification of the lysines. Nevertheless, to limit possible effects of the conjugation reactions on actin polymerization, these were performed under conditions favoring the polymerized state of actin (F-actin). This was the case even in experiments where actin (after completed conjugation reactions) was taken through depolymerization-polymerization cycles before use (Filament Formation Method 1 below).

**Figure 3:**
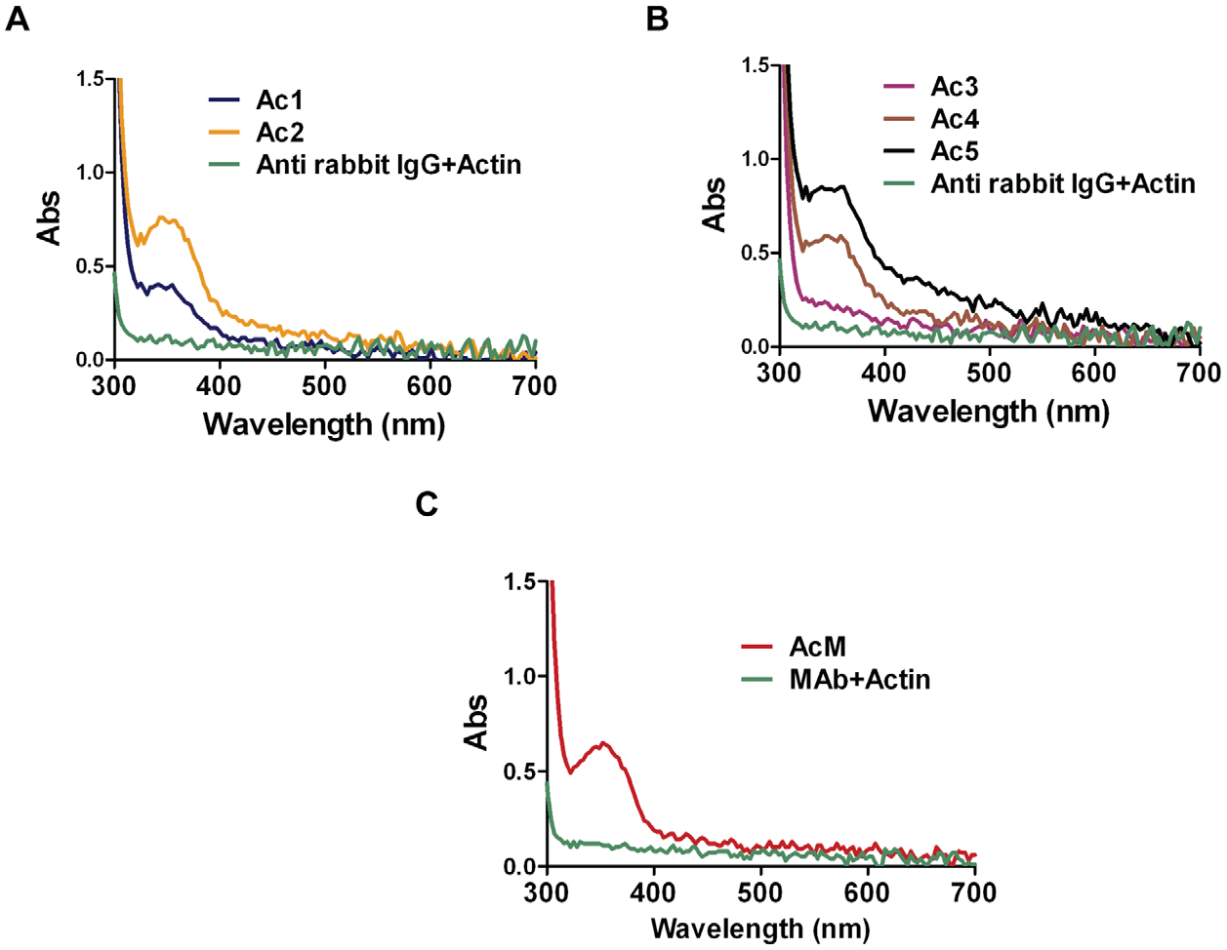
Confirmation of actin-antibody conjugation by absorbance spectroscopy. (**A**) Presence of the UV traceable bis-aryl hydrazone bond verified by measuring absorbance of Ac1 and Ac2 samples in Nano-Drop spectrophotometer (ND-100) with unmodified actin sample (green) as control. Note, the comparatively noisy spectra result from a 1 mm optical path length in the Nano-drop spectrophotometer and multiplication by 10 before plotting to make data comparable to measurements using a conventional spectrophotometer with 1 cm path length. (**B**) Data similar to those in (A) but for Ac3, Ac4 and Ac5 conjugates. (**C**) Data for anti-human CD45 monoclonal antibody (MAb) conjugated with actin (AcM).

Data from semi-quantitative SDS-PAGE (Fig. 2B–D; Table S1) immediately after completion of the conjugation reaction suggested that the fraction of the actin monomers which were conjugated with a-rIgG ranged from 20–40% for the different conjugation schemes (Table S2). Moreover, SDS-PAGE analysis of the pellet (with collected F-actin) after ultracentrifugation, under actin polymerizing conditions suggested that the conjugates were effectively incorporated into filaments as conjugated G-actin would not precipitate (Fig. 2B, lanes 3–4). However, unlike in the microscopy based analyses below we did not know the length distribution for the pelleted actin filaments. Thus, some of these filaments may be short enough (<300 nm) to appear point-like (due to diffraction limitation) in the microscopy based measurements. Bands in the SDS-PAGE, corresponding to antibody-actin conjugates at molecular weights ∼65 and ∼100 kDa (Fig. 2B,C) show that either the light chain or the heavy chain of the antibody are linked to actin in a 1∶1 complex. Bands corresponding to higher molecular weight than 100 kDa demonstrate that higher order complexes were also formed (>1 G-actin/a-rIgG). This is attributed to the fact that the antibodies had 4.6–6.7 cross-linkers on average compared to only 0.4, 1.0 and 1.3 cross-linkers per actin monomer (Table S2). Because our conjugation method produces UV traceable bis-aryl hydrazaone bonds, the conjugation was also verified spectrophotometrically (Fig. 3A–C) a method that, when appropriately calibrated, would be useful for high-throughput quality control, i.e. to verify appreciable attachment of antibodies to the filaments.

It is likely that a fraction of the conjugates formed via the antibody light chain exhibited compromised recognition of antigen (r-IgG in the present work) because of inappropriate orientation of the antigen binding sites (Fab). Similar problems are likely for several of the antibodies which bound to more than one actin monomer.

We found a similar degree of antibody-actin conjugation according to SDS-PAGE and absorbance spectra for monoclonal (CD45; data not shown) and polyclonal (a-rIgG) antibodies (data in Fig. 2B,C and Fig. 3C). This was expected with the current cross-linkers but the finding is important since another type of hetero-bifunctional cross-linker, recently used for antibody conjugation to microtubules [15], could only be applied to polyclonal antibodies.

### Antigen Capture by Antibodies on HMM-bound Actin Filaments

**Figure 4:**
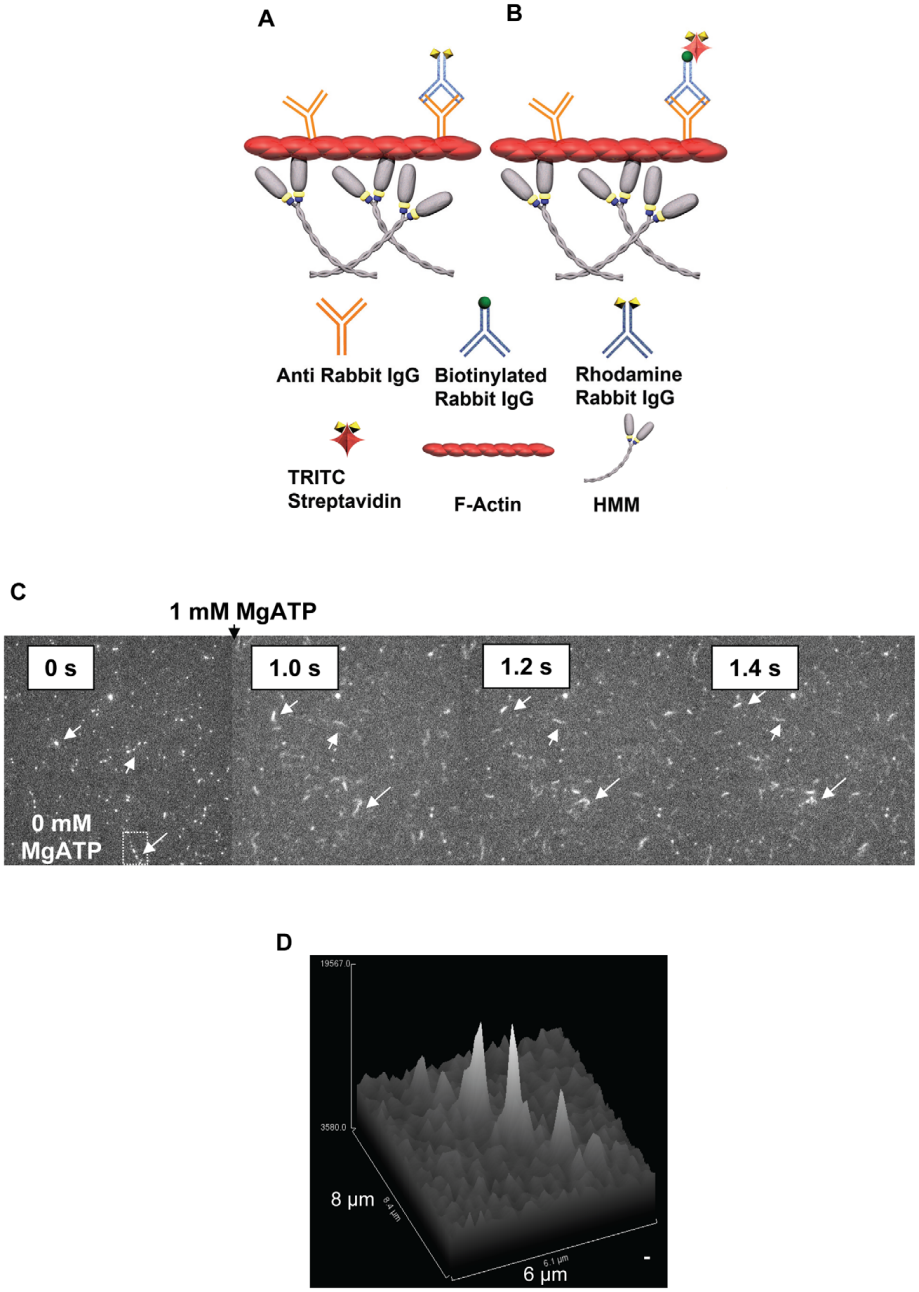
Heavy meromyosin driven transportation of antibody-analyte complexes. (**A–B**) Principles. (**C**) Time series showing fluorescent r-IgG molecules (white spots) where a substantial fraction was bound to a-rIgG on HMM propelled actin filaments. When filaments started to move (second frame from the left), after addition of MgATP, they appeared more elongated and individual fluorescent spots could no longer be seen. Arrows indicate some of the motile filaments (see further Movie S1). The directed sliding at a velocity close to 10 µm s^−1^, and with different winding paths for different filaments, unambiguously shows that the filaments are propelled by HMM rather than transported by directed flow or Brownian motion (cf. [55], [60]). Each frame: 50×50 µm^2^. (**D**) Surface profile plot of intensity within rectangular area in (C), indicating 15 Rh-rIgGs along a 7 µm long filament segment. Motility on TMCS surfaces incubated with HMM (120 µg mL^−1^). Temperature: 28–30°C. Image enhancement by histogram stretching.

Next, we demonstrated capture (Fig. 4) and transportation of fluorescent analytes (antigens) in an in vitro motility assay using HMM to propel the conjugated actin filaments. In these experiments Rh-rIgG was used as the analyte with a-rIgG as the capturing antibody. The actin-(a-rIgG) conjugates were generally composed of actin modified with 1 (Ac1) or 2 (Ac2) fold molar excess of C6-SANH (resulting in 0.4 and 1.0 linkers per G-actin; Table S2) and a-rIgG modified in the presence of 10-fold molar excess of C6-SFB (6.7 linkers/a-rIgG).

In a first set of experiments F-actin was first depolymerized after the conjugation reaction and G-actin monomers were then co-polymerized with un-modified G-actin (1∶3 ratio; “Filament Formation Method 1″) before use in the in vitro motility assay. The co-polymerization was necessary because antibody conjugated G-actin alone, did not polymerize effectively to form filaments. Successful binding of the actin co-polymers to HMM (Fig. 4A,B) on a TMCS derivatized surface was monitored following pre-incubation of the co-polymer (2 µM; total monomer concentration) in solution, with 100 nM Rh-rIgG or (for the monoclonal antibody) fluorescent anti-mouse IgG. Following this procedure, fluorescent spots (Fig. 4C,D) were observed along less than 20% of the HMM-bound actin filaments (Table 1; Movie S1), verifiable due to simultaneous actin filament labeling with fluorescent phalloidin (APh; Movie S2). When conjugated actin filaments had instead been immobilized to HMM in the in vitro motility assay flow cell prior to the incubation (1 h) with 10 nM Rh-rIgG a somewhat higher (35%) fraction of Rh-rIgG labeled filaments was observed but there was also increased non-specific binding of Rh-rIgG to the surface outside the filaments. Importantly, no fluorescent spots due to Rh-rIgG were observed along control filaments without a-rIgG.

**Table 1:** Fraction of APh labeled filaments that were observed to also be labeled with Rh-rIgG or fluorescent secondary antibody to monoclonal CD45 antibody.

Experiment	Fraction labeled(mean ± SEM)	Filament Formation Method	Comment
1	16±2%(n = 990)	1	Incubation in solution (100 nM Rh-rIgG). Observation in a60 assay solution
2	35±3% (n = 440)	1	Incubation in flow cell (10 nM Rh-rIgG), a60
3	16±2%(n = 503)	1	Incubation in solution (100 nM Rh-anti-mouse IgG). Observation in a60 assay solution. Monoclonal antibodies (CD45)
4	35±2%(n = 1196)	2	Incubation in solution (1000 nM Rh-rIgG), aMC130
5	49±4%(n = 382)	2	Incubation in solution(100 nM Rh-rIgG), aMC130

Data are given as mean ± SEM where SEM was estimated from the observed fraction, f, according to SEM = √f(1-f)/n where n was the total number of observed filaments in a given experiment. The data were obtained in assay solution as filament motility made it more straightforward to unequivocally associate all fluorescent molecules with filaments. Polyclonal antibodies (a-rIgG) were used if not otherwise stated.

The co-polymerization of antibody conjugated actin monomers with non-conjugated monomers in a 1∶3 ratio as described above, was expected to reduce the number of antibodies from 70–140 µm^−1^ (expected for a 20–40% conjugation ratio and 360 actin monomers per µm) to 18–35 µm^−1^ in the co-polymer (see further below). In an effort to increase the antibody density, we performed experiments where a-rIgG conjugation of F-actin was not followed by depolymerization-co-polymerization steps. Instead, the conjugated F-actin was kept in solutions favoring the filament state throughout. We denoted this condition “Filament Formation Method 2″. In the latter case, 35–50% (Table 1) of the actin filaments exhibited binding of Rh-rIgG, compared to less than 30% for Filament Formation Method 1. Increasing the Rh-rIgG incubation concentration from 0.1 µM to 1 µM did not appreciably increase the fraction of filaments with bound Rh-rIgG. However, increased density of Rh-rIgG was observed on the motility assay surface either due to non-specific binding of Rh-rIgG or to binding (e.g to HMM) of Rh-rIgG conjugated short actin oligomers or monomers.

Similar Rh-rIgG labeling density along a-rIgG conjugated actin filaments at both 0.1 and 1 µM Rh-rIgG concentrations suggests saturation of Rh-rIgG binding at less than 0.1 µM Rh-rIgG. With a total actin monomer concentration of 2 µM, this implies that less than 5% of the actin monomers display a-rIgGs with Rh-rIgG binding capability, i.e. the average density of functional a-rIgG is less than 18 µm^−1^, compared to a value between 70 and 140 µm^−1^ expected from the SDS-PAGE data (see above).

**Figure 5:**
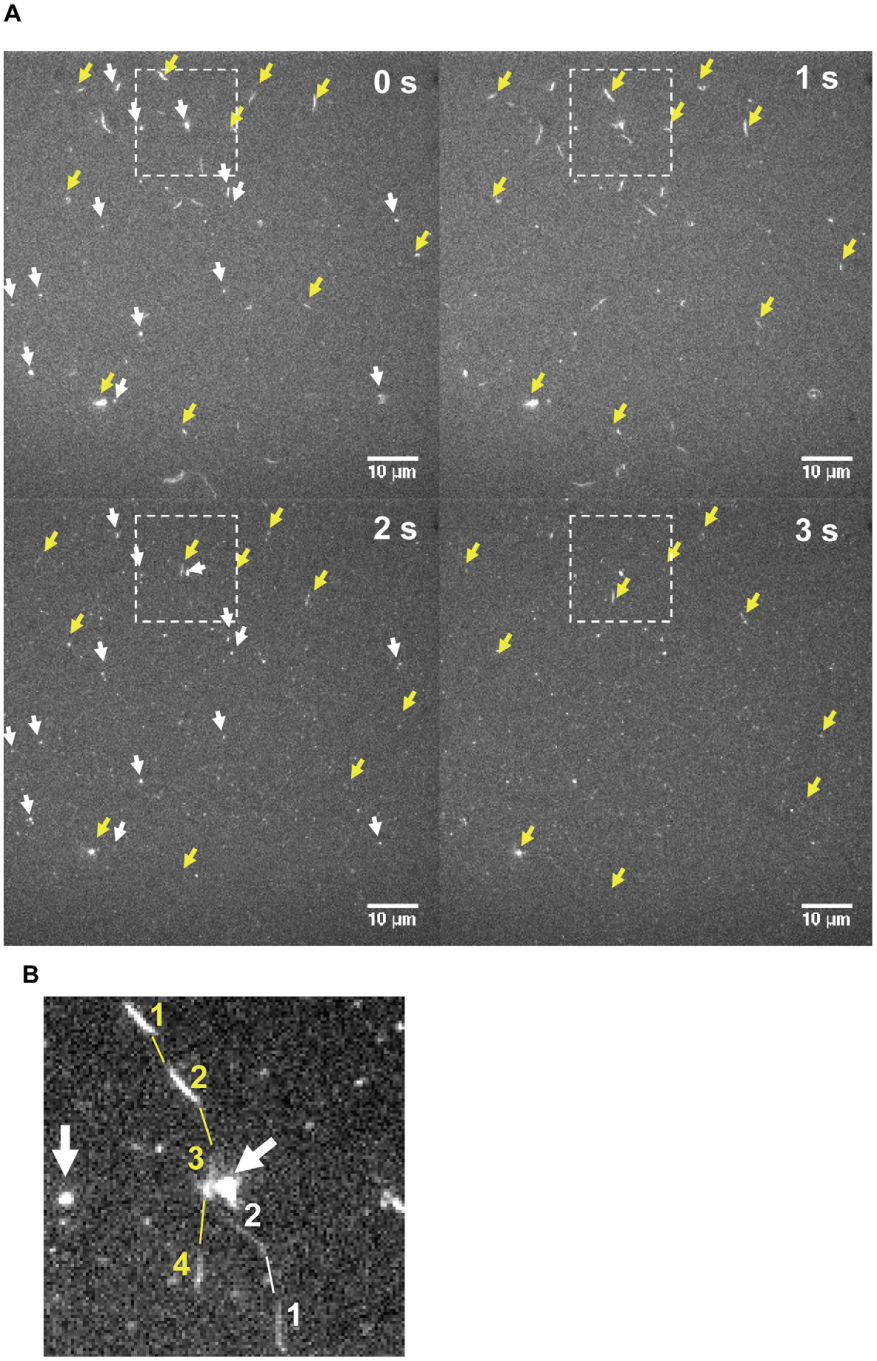
Fluorescence micrographs (0.2 s exposure time) illustrating the fraction of labeled filaments, fraction of motile filaments and variability in degree of Rh-rIgG binding. (**A**) Time series. The images labeled 0 and 1 s were obtained using FITC filter set to observe APh-labeled filaments whereas the images labeled 2 and 3 s were observed using TRITC filter sets to visualize Rh-rIgG binding to filaments. The yellow arrows point at filaments which moved and which could be observed in both filter sets (indicating binding of Rh-rIgG). White arrows point to stationary filaments that could also be observed in both filter sets, whereas filaments without arrows were apparently unlabeled with Rh-rIgG. Images histogram-stretched to map a gray scale level of 25 000 in the original image to 65530 (16 bit) in the processed image. All images were processed similarly. Note that some filaments were very difficult to observe in the TRITC filter set due to low signal-to-noise ratio. Fluorescent spots observed in TRITC filter set but not in the FITC filter set were attributed to surface immobilized Rh-rIgG molecules (or Rh-rIgG aggregates) that were either bound to actin monomers or very short oligomers or not bound to actin. (**B**) Image obtained by summing the part within rectangles for the time series in (A). White arrows: see (A). Lines connecting snapshot images of filament indicate paths for filaments observed only in FITC filter (without Rh-rIgG; white) or in both FITC and TRITC filter sets (with Rh-rIgG, yellow). Starting point of filaments indicated “1″. Filament snapshots in positions 3 and 4 for yellow path represent Rh-rIgG. Fluorescent spots not indicated by arrows were attributed to surface immobilized Rh-rIgG molecules (or Rh-rIgG aggregates) that were either bound to actin monomers or very short oligomers or not bound to actin.

For filaments formed both by Methods 1 and 2, the number of Rh-rIgG molecules was usually less than 6 and only occasionally more than 20 per µm filament length (>3000 Rh-rIgG labeled filaments studied). Observed Rh-rIgG molecules were often concentrated along filament segments of less than 1 µm length, and with Rh-rIgG free segments in between. Finally, several filaments without Rh-rIgG were also observed (Fig. 5).

### HMM Propelled Actin Filaments Effectively Transport Antibody-antigen Conjugates

**Figure 6:**
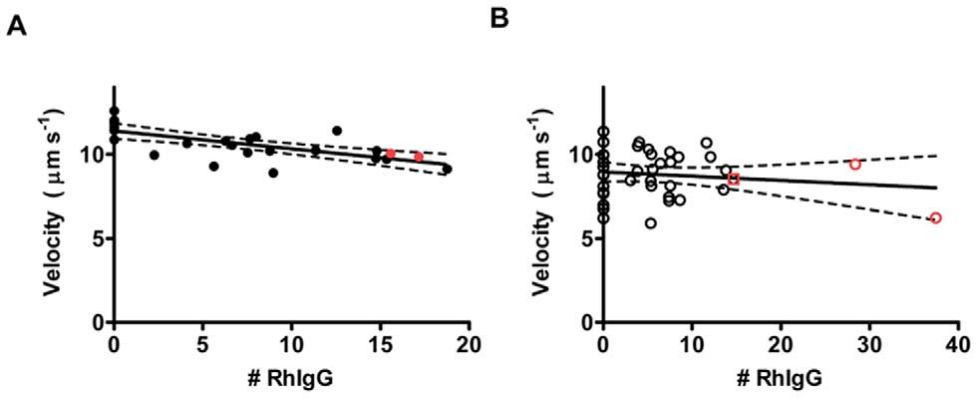
Sliding velocity plotted against the number of Rh-rIgG molecules bound to HMM propelled actin filaments. (**A**) Data obtained in a60 assay solution (Filament Formation Method 1). (**B**) Data obtained in aMC130 solution (Filament formation Method 2). The straight lines obtained by linear regression of all data (black and red symbols) in each figure. Dashed lines represent 95% confidence limits obtained in the regression analysis. Red symbols in A and B: filaments for which the total length of the Rh-rIgG labeled region was less than 1 µm. For one of these filaments (red symbol, 15 Rh-rIgG molecules in (A)), it could also be verified (by observation of APh co-staining) that the actual filament length was less than 1 µm. Blocking actin was not used in these experiments. The number of Rh-rIgG molecules was determined as described in Materials and Methods. This procedure gave approximate values that were not generally integers.

The discrepancy between the SDS-PAGE based estimate of antibody conjugation ratio and the fluorescence microscopy based measurements of Rh-rIgG density along actin filaments suggest a higher total density (µm^−1^) of antibodies (a-rIgG) along an actin filament than indicated by the Rh-rIgG labeling. This idea accords with the above suggested prevention of antigen binding to a fraction of the antibodies immobilized to actin via their Fab fragment (cf. Fig. 2). Therefore, we were concerned about possible filament detachment from HMM upon addition of 1 mM MgATP when the population of myosin heads in strongly actin-attached states is reduced [60]. Consistent with this view, the apparent background fluorescence decreased markedly upon addition of 1 mM MgATP. On the other hand, all Rh-rIgG-labeled objects that were readily identified as filaments generally remained on the surface. However, in the absence of methylcellulose a lower fraction of Rh-rIgG labeled filaments were observed some time after MgATP infusion. These results may be interpreted to mean that the antibodies on actin do indeed compromise myosin binding due to detachment from HMM for very short filament fragments (part of the apparent background fluorescence) and also, occasionally for longer filaments in the absence of methylcellulose. Whereas, a majority of the filaments started to move upon MgATP addition (Fig. 4C; Movie S1) both in a60 and aMC130 solution, the fraction of the filaments that was found to be motile tended to be lower than for non-conjugated normal filaments (Table 2). Also the sliding velocity tended to be reduced by conjugation, but the effect was small and inconsistent (around 10%; Table 2). Similar velocities were observed for the Ac1 (10.01±0.44, n = 14 filaments) and Ac2 conjugates (10.70±0.6, n = 14 filaments) when carrying Rh-rIgG molecules as for non-conjugated filaments in the same experiment (10.01±0.44, n = 12 filaments). Moreover, there was no noticeable difference in motility between Filament Formation Methods 1 and 2, with or without blocking actin (cf. Fig. 6). Results for these different conditions are therefore pooled in Table 2. Motility was observed for all seven studied batches of conjugated actin filaments, although two early experiments were not analyzed in full quantitative detail because the experimental protocol was not fully developed.

**Table 2:** The effect of a-rIgG-Rh-rIgG complexes on actomyosin motility under different conditions.

Condition	Velocity (µm s^−1^)	Fraction of motile (%)
a60 control	10.44±0.48 (n = 4)	0.77±0.06 (n = 4)
a60 conj	9.09±0.57 (n = 4)	0.54±0.07 (n = 4)
a60 difference	−1.35±0.46 (n = 4; p = 0.062)	−0.24. ±0.047 (n = 4; p = 0.0136)*
aMC130 control	10.05±0.73 (n = 5)	0.75±0.03 (n = 3)
aMC130 conj	9.12±0.15 (n = 5)	0.39±0.06 (n = 3)
aMC130difference	−0.92±0.84 (n = 5; p = 0.37)	−0.36. ±0.082 (n = 3; p = 0.0892)

Data given as mean ± SEM. Filaments with different densities of Rh-rIgG pooled in the analysis (cf. Fig. 6). Average velocity based on n experiments (n separate flow cells and experimental occasions). The velocity for each given experiment obtained as mean smooth (CV<0.5) velocity value for 8–19 filament paths. The fraction of motile filaments based on >40 observed filaments per flow cell except for one control experiment where 19 filaments were observed. *Statistical significance (p<0.05). Paired t-test.

Importantly, the sliding velocity did not decrease with increased number (#) of Rh-rIgG molecules per filament (slope, velocity vs # Rh-rIgG: −0.032±0.013 µm s^−1^ (# Rh-rIgG)^−1^; n = 4; p = 0.293). The result was similar for each of four experiments (two illustrated in detail in Fig. 6) obtained in different solutions (a60 and aMC130), in the presence and absence of blocking actin and with filaments formed by Methods 1 and 2.

The few filaments with more than 20 Rh-rIgG molecules demonstrated long-distance motility (>100 µm; throughout the observation period), but with some temporary stops in between periods of sliding at velocities similar to those for non-conjugated filaments. Because a large fraction of the a-rIgG molecules lacked Rh-rIgG binding capability, the above results suggest that actomyosin motility is not significantly inhibited for antibody densities along the actin filament of more than 20 µm^−1^ and possibly up to 50 µm^−1^ (e.g. for a filament with close to 40 Rh-rIgG µm^−1^ in Fig. 6).

Actin filaments with (a-rIG)-rIgG-biotin-streptavidin complexes (Fig. 4B) were also propelled by HMM (Movie S3) after preincubation of Ac1 filaments (2 µM; monomer concentration) with BT-rIgG (10–500 nM) and TRITC-labeled streptavidin (≤50 nM). The number of observed Ac1-(BT-rIgG)-streptavidin complexes was lower than with Rh-rIgG, consistent with low TRITC-streptavidin concentrations (to prevent large aggregate formation). The velocity of motile actin filaments with BT-rIgG-streptavidin complexes (Table 3) was, however, similar or only slightly reduced, compared to that of filaments with Rh-rIgG substantiating the employment of HMM based transport of antibody labeled actin filaments with bulky protein cargoes in future devices.

**Table 3:** The effect of different antibody-antigen complexes on sliding velocity (µm s^−1^) in two different assay solutions.

Sample	a60 (µm s^−1^)	aMC130 (µm s^−1^)
Actin (control)	9.82±0.18 (n = 2)	9.47±0.30 (n = 3)
Actin-(a-rIgG)-(Rh-rIgG)	9.14±0.18 (n = 3)	9.39±0.06 (n = 3)
Actin-(a-rIgG)-(BT-rIgG)-SA	8.64±0.43 (n = 3)	9.34±0.07 (n = 3)

Data given as mean ± SEM. Actin-(a-rIgG)-(Rh-rIgG): Actin conjugated with a-rIgG followed by binding of Rh-rIgG. Actin-(a-rIgG)-(BT-rIgG)-SA: complex with BT-rIgG and TRITC-labeled streptavidin (SA). Average velocity based on n experiments (n separate flow cells and experimental occasions). Velocity in each flow cell estimated as in Table 2 No significant difference between any pair of data according to paired t-test (a60; control data excluded) or repeated measures ANOVA (aMC130). Note: study was not designed for high statistical power since small (<20%) effects are not of relevance for the future use of antibody-labeled filaments in applications.

We also observed similar velocities for complexes of antigens bound to monoclonal antibodies on actin (8.77±0.39 µm s^−1^; n = 21 filaments) as in the absence of conjugates (8.67±0.18 µm s^−1^; n = 15 filaments).

### Limitations in the Antibody-conjugation of Actin Filaments

The highest Rh-rIgG labeling densities (observed for both Methods 1 and 2) were 20–45 Rh-rIgG µm^−1^ corresponding to conjugation of 5–10% of the actin monomers (∼360 µm^−1^; [38]). However, with more than 50% of the filaments apparently unlabelled, and a vast majority of the remaining filaments with less than 6 Rh-rIgG molecules µm^−1^ (<2% of the actin monomers), the results suggest that the Rh-rIgG/actin monomer stochiometric ratio is substantially less than 5% (see above). This is consistent with the saturation level of Rh-rIgG binding to a-rIgG conjugated actin filaments (see above).

In attempts to directly obtain the Rh-rIgG/actin monomer ratio from fluorescence microscopy images, the length of all filaments in an image frame (obtained by Filament Formation Method 2) was first measured for calculation of the total number of actin monomers. Next, after switching filter sets we obtained the total background-subtracted intensity attributed to Rh-rIgG labeling of the filaments. These measurements suggested (assuming 60 IntU per Rh-rIgG, Materials and Methods) that 0.5 and 0.7% of the actin monomers in the two experiments were labeled with Rh-rIgG. However, these values are likely to underestimate the true Rh-rIgG binding. Thus, a fraction of the filaments with bound Rh-rIgG may go undetected in fluorescence microscopy, due to low signal-to-noise ratio (see Materials and Methods), with a limit of detection per diffraction limited spot (diameter ∼250 nm) of 2 fluorophores (corresponding to 8 fluorophores µm^−1^ of the filament length). Thus, our fluorescence microscopy measurements may fail to detect up to 8 fluorophores (∼4 Rh-rIgG molecules; 1% of the actin monomers) per µm of the filament length. Further underestimation of the Rh-rIgG density resulted from photobleaching, causing loss of up to ∼50% of the Rh molecules after 10 s of illumination. Correction for photobleaching now suggests that 1.0–1.4% of the actin monomers are labeled by Rh-rIgG. When also taking into account the limitations of optical detection, the value would be slightly higher, but not more than 5% of the actin monomers. Thus, by also considering the saturation data for Rh-rIgG binding, we are confident to state that between 1 and 5% of the actin monomers in filaments formed by Filament Formation Method 2 (and less for Filament Formation Method 1) display a-rIgG with r-IgG binding capability. This is clearly less than the a-rIgG conjugation according to SDS-PAGE (20–40% of the actin monomers).

The discrepancy between fluorescence microscopy and SDS-PAGE data may partly be attributed to a fraction of a-rIgG molecules without rIgG binding capacity (see above). However, this would not explain the patchy labeling with Rh-rIgG, i.e. with observed clusters, in regions having more than 20 Rh-rIgG µm^−1^ separated by unlabeled segments. Neither would the existence of some a-rIgG without antigen binding capability account for the observation of several µm long filaments without any observed Rh-rIgG labeling. To account for these findings, we hypothesize that actin filaments have an increased tendency to break down into monomers and/or small fragments when conjugated with a-rIgG. The presence of small filament fragments is consistent with the large reduction in background fluorescence upon MgATP addition possibly attributed to detachment, from HMM, of small filament fragments with a-rIgG-(Rh-rIgG) complexes (see above). The initial fragmentation may be followed by re-assembly of new filaments mainly from non-conjugated monomers and fragments. This idea is consistent with the inhibition of actin filament polymerization for low ratios of non-conjugated:conjugated actin monomers. We envisage that not only actin-(a-rIgG)-actin oligomers, but also single a-rIgG conjugated monomers may be rejected in the polymerization process (e.g. due to steric hindrance, slower diffusion etc.) in favor of non-conjugated G-actin (cf. previous results with microtubules [61]). The observation of clusters of fluorescent r-IgG molecules in regions or along some filaments may have a similar basis. Thus, the non-fluorescent regions may be due to initial polymerization of non-conjugated monomers followed by addition of the conjugated monomers in a sequence, when their second-order on-rate constant multiplied by their concentration is higher than for the non-conjugated monomers. It is also possible that several neighboring a-rIgG conjugated monomers break off together from the filaments immediately after conjugation, forming highly conjugated µm long filaments that may then be observed in the fluorescence microscope, possibly after re-annealing to largely non-conjugated filament segments. Additional contribution to clustering of Rh-rIgG could be the fact that certain appropriately immobilized a-rIgG molecules may bind 2 Rh-rIgG. However, this can clearly not explain the presence of more extended (∼1 µm) labeled segments.

To summarize this section: The reasons for low observed Rh-rIgG density (corresponding to 1–5% of actin monomers) along a-rIgG conjugated actin filaments is most likely due to: 1. low stability of filaments or filament fragments with conjugated monomers and reduced tendency for incorporation of such monomers during filament polymerization, and 2. a rather low fraction of actin-attached a-rIgG molecules with Rh-rIgG binding capability. These results suggest that a higher concentration is required for antibody-conjugated than for non-conjugated actin monomers to maintain the polymerized state, i.e. the critical concentration is higher for filament formation from conjugated monomers.

### Summary of Key Findings

We have here, for the first time, used heterobifunctional cross-linkers for covalent attachment of both polyclonal and monoclonal antibodies to cytoskeletal filaments. Importantly we also demonstrated, for the first time, antibody-antigen transportation by the fast actomyosin motor system with minimal effects on motility. This shows that the concerns (see Introduction and [10]) regarding covalent modification of actin filaments and subsequent effective transportation of antibody-antigen complexes were largely unfounded. Accordingly, long-distance transportation was seen even for the most densely Rh-rIgG labeled filaments with velocities similar to non-conjugated filaments and without increased fragmentation during HMM propulsion. With evidence that only a fraction of the a-rIgG molecules bind Rh-rIgG, the transportation of the most heavily Rh-rIgG labeled filaments suggest that, at least 10% of the actin monomers (>40 µm^−1^) may be conjugated with antibodies (e.g. a-rIgG) without appreciable effects on motility. However, our results also showed that main challenges for future work remain. The most important is consistent achievement of high density conjugation of the actin filaments with functional antibodies. Ways forward involve modifications of both the antibody conjugation and filament formation processes. Modifications of the former will require extensive screening of the effect of different cross-linker/protein ratios during the conjugation reaction as well as of other details in the conjugation process. Modification of filament formation would benefit from higher concentrations of conjugated actin monomers and/or higher ratio between conjugated and non-conjugated monomers. Furthermore, it seems clear that better preservation of the polymerized state of actin throughout (from the end of the conjugation reaction, to use of the filaments for transportation of analyte) would be of value.

### Implications of the Results for Biosensing

We found unhindered transportation of almost 20 Rh-rIgG molecules by an actin filament of less than 1 µm length. This compares favorably to less than three covalently attached antibodies per µm of kinesin propelled microtubules [61]. However, our *average* conjugation with functional antibody (a-rIgG) was lower than 20 µm^−1^, and a higher degree of antibody conjugation and transportation was most likely achieved in some recent studies with microtubule-kinesin [13], [14], [16], [62], although direct quantitative measurements were not given. The idea of better cargo-transportation by microtubule-kinesin than by actomyosin is consistent with several arguments related to the different motor characteristics and filament materials properties associated with the two systems (see Introduction and [10]). This idea is also in accord with the findings that several “comparatively large cargoes” have been transported by microtubule-kinesin, e.g. viruses, microspheres etc. [13], [19], and not so readily by HMM propelled actin filaments [21]. However, we showed here that a rather high density of a-rIgG-Rh-rIgG complexes along the actin filament (each likely to extend ∼20 nm from the filament surface) and even higher density of a-rIgG (antibodies) alone, did not appreciably affect actomyosin motility. Nor was motility inhibited by the addition of streptavidin to a-rIgG-rIgG complexes. These results argue strongly against obligate filament rotation around its long axis during sliding [33], [63], one of the factors that has been put forward as a serious challenge [10] for the use of actomyosin in bio-sensing and cargo transportation applications. The present results suggest that actomyosin transport would work very well for protein-sized analytes (e.g. cancer bio-markers such as prostate specific antigens). For “larger” cargoes, e.g. cells, microtubule-kinesin transport may be more effective. However, an interesting alternative in the latter regard is actin bundles formed by the cross-linking via the actin binding protein fascin [21], [32]. These bundles can be transported by HMM at similar velocities as actin filaments, but have considerably larger cargo-carrying capacity.

Our results show that the main limiting factor when using actin filaments in biosensing is not the capability for antibody-antigen transportation per se. The transportation of a few antigens and several more antibodies µm^−1^ (see above) would be sufficient for use in high-sensitivity biosensors aiming to concentrate antigens on a detector site [64], [65], [66].

The main challenge for future work is instead, the consistent achievement of a high density of bioactive antibodies along the actin filaments. If this is achieved, it should be possible to realize the full potential of actomyosin motility for increased separation and concentration speeds as well as for increased miniaturization, due to low flexural rigidity of actin filaments [67], [68]. That such development of practically useful devices is realistic, relies on the present results and on previous developments that have shown guiding of myosin propelled actin filaments with nanoscale precision in bilayer resist channels [22], [69]. The recent demonstration [70], [71] of long-term storage of actomyosin based nanodevices opens for real-world use of actomyosin based separation and diagnostics devices in the near future.

## Supporting Information

Figure S1 **Variability (standard deviation; SD) in intensity values versus mean intensity measured in 10 different frames for each of 6 different filaments in two different experiments (black and red).** Straight lines obtained by regression analysis. Dashed lines represent 95% confidence limits obtained in the regression analysis. For further details, see Methods S1 and Discussion S1.
(TIF)

Table S1 **Describes semi-quantitative analysis of SDS-PAGE.**
(DOC)

Table S2 **Overview of the conjugation reaction.**
(DOC)

Table S3
**Data used to obtain error in the estimated number (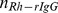) of Rh-rIgG molecules.**
(DOC)

Movie S1 **Actin-antigen complexes before and after addition of MgATP.** Actin filaments (25 nM) conjugated with a-rIgGs adsorbed to a HMM coated surface and visualized by binding of Rh-rIgG (Fig. 4A for schematic illustration). The Rh-rIgGs appear as fluorescent dots that start to move with the filaments when MgATP is added. Same data as in Fig. 4C.
(MOV)

Movie S2 **Aggregation of actin filaments via (a-rIgG)-(Rh-rIgG).** Same flow cell as in Movie S1 but approximately 100 seconds later. All actin filaments are co-stained with Alexa Fluor 488® phalloidin and can therefore be observed after switching from a TRITC to a FITC filter set during the second half of the movie. Aggregation can be seen in the lower left corner in both filters.
(MOV)

Movie S3 **Aggregation of TRITC-streptavidin labeled filaments.** In this set up, analyte (rIgG) molecules (500 nM) are biotinylated and are therefore visualized by binding of TRITC labeled streptavidin molecules (50 nM; cf. Fig. 4B). Bright complexes are seen as evidence of aggregation of filaments via the streptavidin molecules with multiple biotin binding sites.
(MOV)

Methods S1 **Error propagation analysis for estimation of the number of Rh-rIgG molecules from fluorescence microscopy data.**
(DOC)

Discussion S1 **Assumption of similar fluorescence intensity of TRITC-phalloidin on actin and Rhodamine on rabbit IgG.**
(DOC)

Abbreviations S1 **List of abbreviations.**
(DOC)
