# Supplementary figures and images for: Antibodies Covalently Immobilized on Actin Filaments for Fast Myosin Driven Analyte Transport
Source: PLoS One. 2012 Oct 3;7(10):e46298. doi: 10.1371/journal.pone.0046298 (PMC3463588; doi:10.1371/journal.pone.0046298)

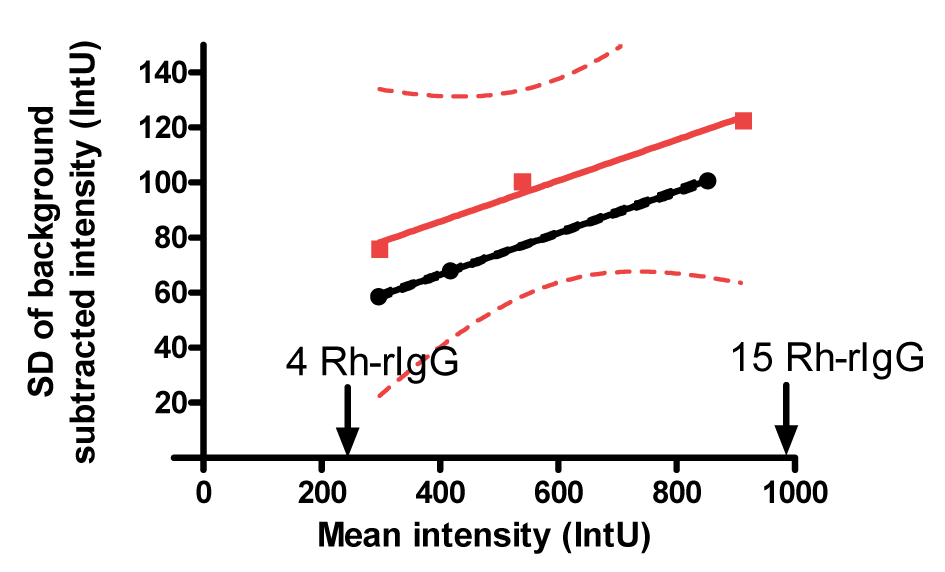

Supplement: Figure S1 — Variability (standard deviation; SD) in intensity values versus mean intensity measured in 10 different frames for each of 6 different filaments in two different experiments (black and red). Straight lines obtained by regression analysis. Dashed lines represent 95% confidence limits obtained in the regression analysis. For further details, see Methods S1 and Discussion S1. (TIF) [file pone.0046298.s001.tif]
