# Supplementary material for: Antibodies Covalently Immobilized on Actin Filaments for Fast Myosin Driven Analyte Transport
Source: PLoS One. 2012 Oct 3;7(10):e46298. doi: 10.1371/journal.pone.0046298 (PMC3463588; doi:10.1371/journal.pone.0046298)
Supplement: Table S1 — Describes semi-quantitative analysis of SDS-PAGE. (DOC) [file pone.0046298.s002.doc]

**Table S1. Semi-quantitative analysis of SDS-PAGE.**

| **Sample name** | **Conjugated actin** | **Free actin** | **Total actin** | **Yield (%)** |
| --- | --- | --- | --- | --- |
| Ac1 | 8.1 | 22.5 | 30.6 | 26.4 |
| Ac2 | 10.9 | 16.6 | 27.5 | 39.4 |
| Ac3 | 5.7 | 16.0 | 21.7 | 26.3 |
| Ac4 | 2.6 | 18.8 | 13.5 | 19.7 |
| Ac5 | 3.4 | 7.1 | 10.5 | 32.5 |

All concentrations are in µM.
