# Supplementary material for: Antibodies Covalently Immobilized on Actin Filaments for Fast Myosin Driven Analyte Transport
Source: PLoS One. 2012 Oct 3;7(10):e46298. doi: 10.1371/journal.pone.0046298 (PMC3463588; doi:10.1371/journal.pone.0046298)
Supplement: Table S2 — Overview of the conjugation reaction. (DOC) [file pone.0046298.s003.doc]

**Table S2.** Overview of the conjugation reaction.

| **Sample name** | **Actin:C6-SANH** | **MSR actin** | **a-rIgG:C6-SFB** | **MSR a-rIgG** | **a-rIgG:actin** |
| --- | --- | --- | --- | --- | --- |
| Ac1 | 1:1 | 0.4 | 1:10 | 6.7 | 1:2 |
| Ac2 | 1:2 | 1.0 | 1:10 | 6.7 | 1:2 |
| Ac3 | 1:1 | 0.4 | 1:5 | 4.6 | 1:2 |
| Ac4 | 1:2 | 1.0 | 1:5 | 4.6 | 1:2 |
| Ac5 | 1:3 | 1.3 | 1:5 | 4.6 | 1:2 |
| AcM# | 1:2 | 1.0 | 1:10# | 6.1# | 1:2# |

Molar substitution ratios (MSR; molar ratio of cross-linker to protein) are shown for the actin modified with C6-SANH and antibodies (a-rIgG or MAb) modified with C6-SFB. **#**Anti-human CD45 monoclonal antibody (MAb). Actin conjugated with MAb (AcM).
