# Supplementary material for: Antibodies Covalently Immobilized on Actin Filaments for Fast Myosin Driven Analyte Transport
Source: PLoS One. 2012 Oct 3;7(10):e46298. doi: 10.1371/journal.pone.0046298 (PMC3463588; doi:10.1371/journal.pone.0046298)
Supplement: Table S3 — Data used to obtain error in the estimated number () of Rh-rIgG molecules. (DOC) [file pone.0046298.s004.doc]

**Table S3.** Data used to obtain error in the estimated number (
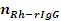
) of Rh-rIgG molecules.

| **Measured intensity (I; main Eq. 1)** | **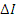** | **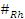** | **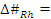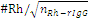** | **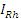** | **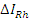** | **nRh-rIgG** | **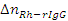** |
| --- | --- | --- | --- | --- | --- | --- | --- |
| 120 | 60 | 1.9 | 1.85 | 28.9 | 3.6 | 2 | 1.8 |
| 300 | 70 | 1.9 | 1.31 | 28.9 | 3.6 | 5 | 2.7 |
| 600 | 90 | 1.9 | 0.83 | 28.9 | 3.6 | 10 | 3.8 |
| 900 | 110 | 1.9 | 0.59 | 28.9 | 3.6 | 15 | 4.8 |
| 1200 | 130 | 1.9 | 0.48 | 28.9 | 3.6 | 20 | 5.6 |
