# Supplementary material for: Antibodies Covalently Immobilized on Actin Filaments for Fast Myosin Driven Analyte Transport
Source: PLoS One. 2012 Oct 3;7(10):e46298. doi: 10.1371/journal.pone.0046298 (PMC3463588; doi:10.1371/journal.pone.0046298)
Supplement: Methods S1 — Error propagation analysis for estimation of the number of Rh-rIgG molecules from fluorescence microscopy data. (DOC) [file pone.0046298.s008.doc]

**Methods S1. Error propagation analysis for estimation of the number of Rh-rIgG molecules from fluorescence microscopy data**

The background-subtracted intensity, I, for Rh-rIgG bound to actin filaments was calculated using Equation 1 in the main paper. The variability (standard deviation) of I between measurements was deduced from repeated intensity measurements of given Rh-rIgG labeled filaments as these were propelled by HMM over motility assay surfaces. Using a given filament for the repeated measurements ensured constant number (
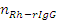
) of Rh-rIgG and short measurement time (~2 s) made photobleaching negligible. Moreover, the fact that the filaments were motile (velocity ~10 µm/s) ensured that the measurements were made with different background intensity profiles in each new frame. Results showing the variation of the standard deviation of the intensity with the mean intensity value are illustrated for two experiments (three filaments in each) in Fig. S1. For each filament the average intensity and standard deviation was estimated from the filament intensity in 10 different locations. It was ensured that no other filaments were included in regions of interest analyzed. Data approximated from Fig. S1 employed in the error propagation analysis are given in Table S3.

The uncertainty (ΔI_Rh_) in the fluorescence intensity (I_Rh_) for a given rhodamine molecule was obtained from measurements of the fluorescence intensity per TRITC-phalloidin molecule when TRITC-phalloidin was assumed to fully occupy its binding sites on actin filaments (see main paper). Here repeated measurements gave an average value of 28.9 IntU with error estimate for this average of 3.6 IntU (SEM; ΔI_Rh_).

Further, in the error propagation analysis the average number (#_Rh_) of rhodamine molecules per rIgG was taken as 1.9 and the uncertainty in this number (SEM: Δ#_Rh_ =
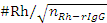
) was assumed to vary as indicated in Table S3 (for rationale, see main paper).

Table S3 summarizes all data used for error propagation analysis to obtain the uncertainty in the estimated value of
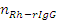
. The latter error estimate, obtained using equation S1, is also given in Table S3.


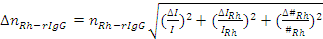
 (Equation S1)
