# Supplementary material for: Antibodies Covalently Immobilized on Actin Filaments for Fast Myosin Driven Analyte Transport
Source: PLoS One. 2012 Oct 3;7(10):e46298. doi: 10.1371/journal.pone.0046298 (PMC3463588; doi:10.1371/journal.pone.0046298)
Supplement: Discussion S1 — Assumption of similar fluorescence intensity of TRITC-phalloidin on actin and Rhodamine on rabbit IgG. (DOC) [file pone.0046298.s009.doc]

**Discussion S1. Assumption of similar fluorescence intensity of TRITC-phalloidin on actin and Rhodamine on rabbit IgG.**

In the analysis of fluorescence microscopy data to obtain the number of Rh-rIgG molecules on actin filaments, it was assumed that rhodamine molecules (Rh) on rIgG and TRITC-phalloidin (TRITC-Ph) on actin exhibit similar fluorescence intensity. This idea is supported by several lines of argumentation. Thus, if the intensity per fluorophore had been higher for Rh on IgG than for TRITC-Ph on actin, the highest possible, and also most likely value for Rh would be in the range 60-100 IntU, to account for the main peak in the intensity distribution (Fig. 1D) and the observed photoblinking of about 70 IntU (Fig. 1B-C). However, this interpretation would mean that a majority of the IgGs would have just 1 Rh-rIgG because most observed single fluorescence spots had intensities in the range 60-100 IntU (Fig. 1D) rather than between 120 and 200 IntU (corresponding to two rhodamines). This idea is clearly inconsistent with the spectrophotometric data suggesting 1.9 Rh molecules per IgG on average. Moreover, if the intensity per Rh had been 60-100 IntU one would expect well discriminated peaks in the intensity distribution, one in the range 60-80 IntU and one in the range 120-200 IntU. Instead there was one single broad peak between 60 and 140 IntU, with three apparent and partly overlapping sub-peaks separated by approximately 30 IntU. In view of the above arguments, the alternative with ~30 IntU per Rh (just as for TRITC-Ph) seems considerably more likely than the higher value excluding any underestimation of the number of Rh-rIgG molecules per actin filament.

The other possibility to consider is that the intensity per Rh on IgG was actually lower than for TRITC-Ph on actin. If this was the case our data have underestimated the number of Rh-rIgG per actin filament length. However, it seems unlikely that photoblinking of an apparently single spot with intensity 70 IntU can be explained if the intensity/Rh is considerably lower than 30 IntU (say down to 20 IntU) as it is unlikely that two fluorophores switch off and then on again simultaneously. In contrast, blinking of one fluorophore (out of two on rIgG) with intensity around 30 IntU may lead to masking of the remaining fluorophore in the background with its pixel to pixel standard deviation of ~30 IntU above the mean background level (see main paper).

The above argumentation lends support to the assumption that the fluorescence intensity per Rh molecule bound to IgG is very similar to the fluorescence intensity of TRITC-Ph bound to actin. It is under this assumption that we perform the error propagation analysis above.
