# Supplementary material for: Antibodies Covalently Immobilized on Actin Filaments for Fast Myosin Driven Analyte Transport
Source: PLoS One. 2012 Oct 3;7(10):e46298. doi: 10.1371/journal.pone.0046298 (PMC3463588; doi:10.1371/journal.pone.0046298)
Supplement: Abbreviations S1 — List of abbreviations. (DOC) [file pone.0046298.s010.doc]

**Abbreviations S1. List of abbreviations**

Ac - Actin conjugated with anti rabbit IgG

AcM - Actin conjugated with monoclonal antibody

a-rIgG - Anti rabbit IgG

APh - Alexa Fluor 488® phalloidin

ATP - Adenosine-5'-triphosphate

BCA- Bicinchoninic acid

BSA - Bovine serum albumin

BT-rIgG - Biotinylated rabbit IgG

C6-SANH - C6-succinimidyl 6-hydrazinonicotinate acetone hydrazone

C6-SFB - C6-succinimidyl 4-formylbenzoate

DH_2_O - Deionized water

DMSO - Dimethylsulfoxide

DTT - Dithiothreitol

EGTA - Ethylene glycol tetraacetic acid

EMCCD - Electron multiplying charge coupled device

Fab - Antigen binding site

FITC - Fluorescein isothiocyanate

HABA - 4′-Hydroxyazobenzene-2-carboxylic acid

HEPES - (4-(2-hydroxyethyl)-1-piperazineethanesulfonic acid)

2-HP - 2-hydrazinopyridine.dihydrochloride

HMM - Heavy meromyosin

IVMA - In vitro motility assay

MAb – Monoclonal antibody

MES - 2-(N-morpholino)ethanesulfonic acid

MOPS - 3-morpholinopropane-1-sulfonic acid

MSR - Molar substitution ratio

PBS - Phosphate buffered saline

Rh-rIgG - Rhodamine labeled rabbit IgG

Rh - Rhodamine

RhPh - Rhodamine phalloidin

rIgG - Rabbit IgG

RT - Room Temperature

2-SBA- 2-sulfobenzaldehyde

SD - Standard deviation

SDS-PAGE - Sodium dodecyl sulphate-polyacrylamide gel electrophoresis

SEM – Standard error of mean

TMCS – Trimethylchlorosilane

TRITC - Tetramethylrhodamine isothiocyanate

TLCK - 1-chloro-3-tosylamido-7-amino-2-heptanone
